# Dopaminergic Neuronal Imaging in Genetic Parkinson's Disease: Insights into Pathogenesis

**DOI:** 10.1371/journal.pone.0069190

**Published:** 2013-07-23

**Authors:** Alisdair McNeill, Ruey-Meei Wu, Kai-Yuan Tzen, Patricia C. Aguiar, Jose M. Arbelo, Paolo Barone, Kailash Bhatia, Orlando Barsottini, Vincenzo Bonifati, Sevasti Bostantjopoulou, Rodrigo Bressan, Giovanni Cossu, Pietro Cortelli, Andre Felicio, Henrique B. Ferraz, Joanna Herrera, Henry Houlden, Marcelo Hoexter, Concepcion Isla, Andrew Lees, Oswaldo Lorenzo-Betancor, Niccolo E. Mencacci, Pau Pastor, Sabina Pappata, Maria Teresa Pellecchia, Laura Silveria-Moriyama, Andrea Varrone, Tom Foltynie, Anthony H. V. Schapira

**Affiliations:** 1 Department of Clinical Neurosciences, Institute of Neurology, University College London, London, United Kingdom; 2 Regional Genetics Unit, Department of Clinical Genetics, Birmingham Women’s Hospital, Birmingham, United Kingdom; 3 Department of Neurology, National Taiwan University Hospital, College of Medicine, Taipei, Taiwan; 4 Department of Nuclear Medicine, National Taiwan University Hospital, College of Medicine, Taipei, Taiwan; 5 Instituto Israelita de Ensino e Pesquisa Albert Einstein, Hospital Israelita Albert Einstein, São Paulo, São Paulo, Brasil; 6 Parkinson's and Movement Disorders Unit, Department of Neurology, Hospital Universitario Insular de Gran Canaria, Las Palmas de Gran Canaria, Spain; 7 Center for Neurodegenerative Diseases, University of Salerno, Fisciano Province of Salerno, Italy; 8 Sobell Department of Motor Science, Institute of Neurology, University College London, London, United Kingdom; 9 Department of Neurology, Universidade Federal de São Paulo, São Paulo, São Paulo, Brazil; 10 Department of Clinical Genetics, Erasmus Medical Centre, Rotterdam, The Netherlands; 11 Third Department of Neurology, G. Papanikolaou Hospital, Aristotle University of Thessaloniki, Thessaloniki, Greece; 12 Neurology Service and Stroke Unit, General Hospital S. Michele AOB G. Brotzu, Cagliari, Italy; 13 Department of Biomedical and Neuromotor Sciences, University of Bologna, Bologna, Italy; 14 Division of Movement Disorders, Universidade Federal de São Paulo (UNIFESP), Escola Paulista de Medicina (EPM), São Paulo, São Paulo, Brazil; 15 Department of Molecular Neuroscience, UCL Institute of Neurology, London, United Kingdom; 16 Neurogenetics Laboratory, Division of Neurosciences, Center for Applied Medical Research, University of Navarra, Pamplona, Spain; 17 Institute of Biostructure and Bioimaging, CNR, Naples, Italy; 18 Karolinska Institutet, Department of Clinical Neuroscience, Centre for Psychiatry Research, Stockholm, Sweden; Mayo Clinic, United States of America

## Abstract

**Objectives:**

To compare the dopaminergic neuronal imaging features of different subtypes of genetic Parkinson's Disease.

**Methods:**

A retrospective study of genetic Parkinson's diseases cases in which DaTSCAN (123I-FP-CIT) had been performed. Specific non-displaceable binding was calculated for bilateral caudate and putamen for each case. The right:left asymmetry index and striatal asymmetry index was calculated.

**Results:**

Scans were available from 37 cases of monogenetic Parkinson's disease (7 *glucocerebrosidase (GBA)* mutations, 8 *alpha-synuclein*, 3 *LRRK2*, 7 *PINK1*, 12 *Parkin*). The asymmetry of radioligand uptake for Parkinson's disease with *GBA* or *LRRK2* mutations was greater than that for Parkinson's disease with *alpha synuclein, PINK1* or *Parkin* mutations.

**Conclusions:**

The asymmetry of radioligand uptake in Parkinsons disease associated with *GB*A or *LRRK2* mutations suggests that interactions with additional genetic or environmental factors may be associated with dopaminergic neuronal loss.

## Introduction

Parkinson's disease (PD) is the second most common neurodegenerative disorder worldwide with a prevalence of 1–2% in the population aged over 65 years [1]. The vast majority of PD is late onset, sporadic and of unknown aetiology. However, 5–10% of PD cases are associated with mutations in a range of genes inherited in an autosomal dominant, autosomal recessive or apparently sporadic pattern [2]. The most common genetic risk factor for PD is mutation of the *glucocerebrosidase (GBA)* gene, which encodes a lysosomal enzyme deficient in the lysosomal storage disorder Gaucher disease (GD) [1]. In sporadic PD between 5–30% of cases have a heterozygous *GBA* mutation, with the highest frequency in Ashkenazi Jews, while 8.4% of European autosomal dominant PD families have *GBA* mutations [3]. The second most common genetic cause of PD is the G2019S mutation of *LRRK2,* which typically presents with late onset features resembling sporadic PD [4]. The frequency of *LRRK2 G2019S* in sporadic PD in Caucasians varies from 0.5–5% depending on population studied [1], [4]. Mutations of the *SNCA* gene, which encodes alpha synuclein, the principle component of the Lewy body, cause early onset autosomal dominant PD [5]. *SNCA* mutations are rare, accounting for less than 0.5% of PD cases [1], [5]. The penetrance for development of PD varies greatly between these different genes; being 15% by age 80 for *GBA* [12], around 80% by age 80 for *LRRK2* [4] and almost 100% for *SNCA* mutations [1], [5]. This suggests that mutations in *GBA* or *LRRK2* are not sufficient by themselves to cause symptomatic PDneurodegeneration and that interactions between GBA or LRRK2 protein and other genetic or environmental factors influence penetrance.

Autosomal recessive Juvenile Parkinson's disease (ARJPD) is clinically distinct from both sporadic PD and the genetic causes outlined above [6]. Fifty percent of familial and 15% of sporadic early onset PD (onset before 45 years old) is due to bi-allelic mutations (i.e. homozygous or compound heterozygous) in the *Parkin* gene [1], [6]. *Parkin* disease differs from non-*Parkin* PD in that early lower limb dystonia and bilateral motor Parkinsonian signs are more common, non-motor symptoms are less severe and, in general, there is an absence of Lewy bodies histologically [6], [7], [8]. *PINK1* mutations are the second most common cause of early onset PD, present in 2–4% of cases [9]. Clinical reports of *PINK1* associated PD emphasise a slow disease progression, prominent gait impairment and marked neuropsychiatric symptoms compared to sporadic PD [9]. Mutations in DJ-1 are rare, accounting for just 1% of early onset PD [1], [6]. Bi-allelic mutations in *PINK1, Parkin* and *DJ-1* are highly penetrant for PD, suggesting that interactions with additional genetic or environmental factors are not required for neurodegeneration to occur in these genetic subtypes of PD.

PD is diagnosed clinically based upon the Queens Square Brain Bank criteria, with a positive predictive value of 90% for neuropathological evidence of PD if these criteria are met [1]. In clinical practice imaging to detect pre-synaptic dopaminergic neuronal dysfunction is performed to aid diagnosis when Parkinsonian signs are present but clinical criteria for PD are not fully met [10]. These imaging studies involve visualisation of radioactive dopamine transporter (DAT) ligands (e.g. 123-I-FP-CIT, 99m-Tc-TRODAT-1) or 18F-DOPA with positron emission tomography (PET) or single photon emission computed tomography (DAT-SPECT). DAT-SPECT and 18F-DOPA-PET have been studied extensively in sporadic PD and “Parkinson's plus” syndromes [10]. However, there are limited reports of dopaminergic neuronal imaging in genetic PD. We sought to examine the hypothesis that mutations in *GBA* and *LRRK2* would have asymmetrical loss of radioligand, reflecting initially focal neurodegeneration due to interactions with additional endogenous or exogenous pathogenic factors, whilst PD with bi-allelic *PINK1* or *Parkin* mutations would manifest more symmetrical radioligand loss since mutations in these genes alone is sufficient to induce neurodegeneration. To do this, we studied 123-I-FP-CIT (DaTSCAN) appearances in what is to our knowledge the largest series of genetic PD patients reported.

## Materials and Methods

We formed a consortium of Movement Disorders and Neurogenetics centres from Western Europe, South America and Asia to identify a large series of monogenetic PD cases. Mutations in *GBA*, *SNCA*, *LRRK2*, *Parkin*, or *PINK1* were identified by molecular genetic sequencing according to standard clinical protocols. From amongst such patients individuals who had been imaged with DaTSCAN as part of their initial clinical evaluation were identified by retrospective chart review. Electronic copies of each scan were collated for analysis at a single site (University College London).

A region of interest (ROI) analysis was performed using Image J (NIH, Bethesda, MD). Four ROIs were anatomically defined: right and left caudate, right and left putamen. ROIs were manually defined using the ellipse tool in Image J. To permit calculation of background signal a region of occipital cortex was designated as a 5^th^ ROI. The outcome measure was the specific-to-nondisplaceable binding ratio V3” (ROIstriatum – ROIoccipital/ROIoccipital) [11]. For each ROI, 4 transaxial slices were analysed. Four slices showing the most intense radiotracer uptake were selected for analysis from each case. For each case the right:left asymmetry index (ASI, most severely affected ROI V3”/least severely affected ROI V3”, e.g. ipsilateral caudate ROI V3”/contralateral caudate ROI V3”) was calculated for caudate and putamen [11]. The whole striatal asymmetry index was also calculated (SASI: (ipsilateral-contralateral striatum V3”)/((ipsilateral+contralateral striatumV3”)/2)*100%), where the striatum was defined as the caudate plus putamen value [9]. The ASI calculates the ratio of ligand binding in the most severely affected caudate or putamen compared to the least severely affected side, a ratio of 1.0 indicates perfect symmetry while values of less than 1.0 indicate progressively worse asymmetry. This score enables differentiation of whether asymmetry is predominantly due to loss of caudate or putamen uptake. The SASI gives a summary measure of asymmetry in the whole striatum, defined as uptake in caudate plus putamen. Higher SASI values indicate greater degrees of asymmetry. Images were analysed blind to the genetic diagnosis by a single investigator (AM), apart from the *SNCA* scans which were reported by SB.

Statistical analysis was performed with PASW 20.1 (version 20.1, IBM). Differences in median ASI and SASI between genotypes were sought using the Mann-Whitney U-test. Significance was taken at the 5% level (p = 0.05), with bonferroni correction (i.e. p = 0.05/n where n = number of comparisons). Demographic variables of patients were compared using one way ANOVA and chi-squared test.

### Ethics Statement

All participating institutions had research ethics board approvals for the project and written informed consent was taken as appropriate. No participant had cognitive impairment which meant they could not give informed consent. London: North West London Research Ethics Committee (REC number 10/H0720/21). Spain: The Institutional Review Board (IRB) at the University of Navarra, (Pamplona, Spain) approved the study. Brazil: Ethics committee of Hospital Israelita Albert Einstein Taiwan: Research Ethics committee C. Taiwan: National Taiwan University Hospital. Italy: ethics committee of University of Naples Federico II. Rotterdam: medical ethics committee of Erasmus MC Rotterdam.

## Results

Clinical and genetic characteristics of the 37 genetic PD patients enrolled are given in Table 1. Table 2 summarises DaTSCAN findings. Control DaTSCAN images were available from 12 individuals without neurological disease from the European Nuclear Medicine Consortium (provided by Dr John Dickson, Nuclear Medicine Department University College London Hospital). All scans were performed according to standard clinical protocols. All of the DaTSCANS utilised in the current study had previously been reported as showing reduction of radioligand binding compatible with PD. Ethical approvals were obtained at each centre and written informed consent taken as required. The *PINK1* [9], heterozygous *GB*A mutation associated PD [12], Brazilian *Parkin* and *LRRK2* patients [13], *SNCA* cases [14] and Italian *Parkin* cases [15] have been reported previously.

**Table 1:** Clinical, genetic and demographic characteristics of patient cohort.

	GBA	PARKIN	PINK1	LRRK2	SNCA
Male	7/7(100%)	9/12 (75%)	5/7(55%)	3/3 (100%)	5/8 (62.5%)
Age at scan(years)	50+/−13	44+/−14	42+/−17	51.5+/−19	47.1+/−7.5
Disease duration(years)	7+/−4	14.5+/−10	12.3+/−11	5.5+/3	7.4+/−2
Mutation	N370S/L444P (1/7),N370S (1/7),L444P (1/7),IVS2+1G>A(1/7),E326K(1/7),T369M (1/7),R496H (1/7).	Del exon 3/del exon 3 (1/12),Del exon 3–4/del exon 3–4 (1/12),Del exon 3/del exon 2–3 (1/12),Del exon 2/del exon 2–4 (1/12),C820T/del exon 2 (1/12),G96C/C1305T (1/12),R42P/R42P (3/12),G429EfsX5/G429EfsX5 (3/12).	1573insTTAG/1573insTTAG (1/7),1488+1G>A/1488+1G>A (1/7),1488+1G>A/1252_1488del (5/7)	G2019S (2/3)R1441C (1/3)	G209A (8/8)
L-DOPA use	4/7 (57%)	8/12 (66%)	5/9 (55%)	2/3 (66%)	8/8 (100%)
UPDRS III	29.8+−5	28.2+/−12.7	12.8+/−6	30+/−13	36.2+/−14

**Table 2:** Asymmetry indices for each subtype of genetic Parkinson's disease.

	*GBA*	*LRRK2*	*Parkin*	*PINK1*	*SNCA*
CASI	**0.42** * **[0.33–0.56]**	1.0 [0.6–1.1]	0.88 [0.7–0.93]	1.0 [0.89–1.13]	1.02 [0.9–1.13]
ASI	**0.18** * **[0.08 0.33]**	**0.49** * **[0.13–0.5]**	0.69 [0.2–1.0]	1.05 [0.9–1.4]	1.05 [0.92–1.22]
SASI	**80+/−5.1**	**44+/−11.5**	32+/−6	13.6+/−4.4	13.4+/−11

Key: CASI, PASI = right/left asymmetry index quoted as median and interquartile range for caudate and putamen, SASI = striatal asymmetry index quoted as mean and standard error.

* = significantly different than ASI from control (Mann-Whitney u-test).

The age at assessment (one way ANOVA, p>0,05), sex and proportion of patients taking levo-dopa for each genetic subgroup did not differ significantly (both chi-squared test, p>0.05) (Table 1). The disease duration did not differ between groups (one way ANOVA, F = 2.3, p = 0.076). The UPDRS part III scores for the PINK1 group were significantly lower than the other genetic subgroups (p<0.05 for all comparisons with Bonferroni correction). The control group were all of Western European ancestry but did not differ significantly from the PD group for age (52+/−12 years, p>0.05) or sex (7/12 male, chi-squared test p>0.05).

**Figure 1:**
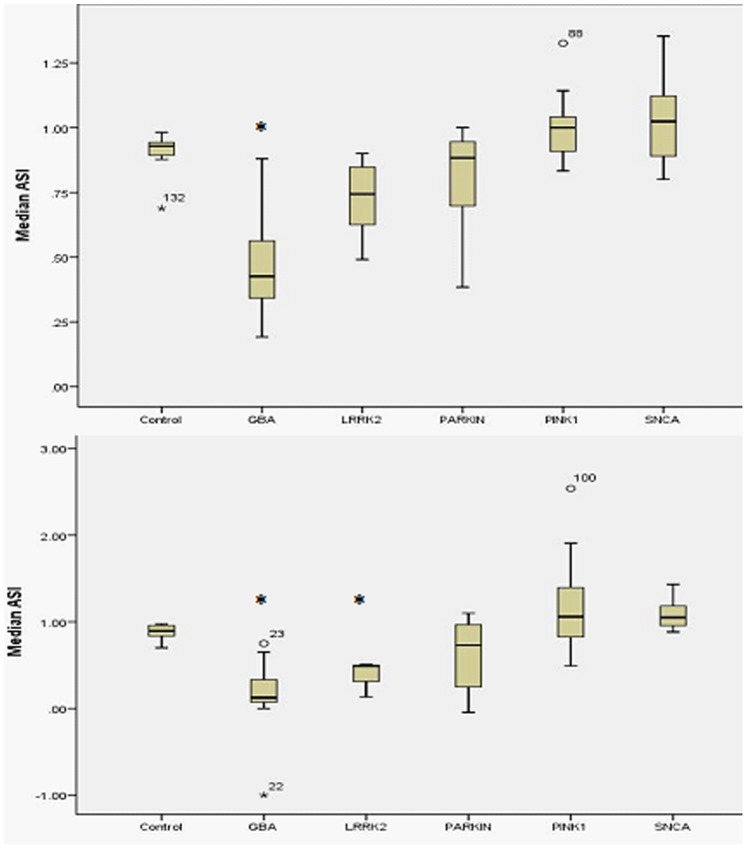
Caudate and putament asymmetry index for subtypes of genetic Parkinson's disease. 1a. Boxplot of caudate asymmetry index for each genotype. Median caudate asymmetry is signficantly different for GBA compared to control. 1b. Boxplot of putamen asymmetry index for each genotype. Median putamen asymmetry is significantly different for GBA and LRRK2 groups compared to control.

The caudate ASI for the PD cases with *GBA* mutations (Mann-Whitney U-test, median ASI 0.42 [0.33–0.56], p<0.001) was different to the control value of 0.92 (interquartile range 0.89–0.92) (Table 1 and Figure 1a). The caudate asymmetry indices for PD with bi-allelic *Parkin* (median 0.88 [0.72–0.93]. p = 0.19), *PINK-1* (median 1.0 [0.9–1.13], p = 0.065), *LRRK2 G2019S* (median 1.0 [0.6–1.1], p = 0.38), or *SNCA* (median 1.02 [0.9–1.13], p = 0.91) did not differ from the control value. This indicates an asymmetric reduction of radioligand uptake in the caudate of PD associated with *GBA* mutations with a relatively symmetrical reduction in ligand uptake in the caudate of *PINK1*, *Parkin, LRRK2 G2019S* and *SNCA* mutation associated PD.

The putamen ASI for PD cases with *GBA* mutations (median 0.18 [0.08–0.33], p<0.001) and *LRRK2* (median 0.52 [0.13–0.5], p<0.001) differed significantly from the control value (median 0.89 [0.82–0.96])(Figure 1b). The putamen ASI for bi-allelic *Parkin* (median 0.69 [0.2–1.0], p = 0.2), *PINK1* (median 1.05 [0.9–1.4], p = 0.14) and *SNCA* (median 1.05 [0.92–1.22], p = 0.30) mutation associated PD did not differ from control values (median 0.89 [0.82–0.86]). This indicates an asymmetric reduction of radioligand uptake in the putamen of PD associated with *GBA* and *LRRK2* mutations with a relatively symmetrical reduction in ligand uptake in the putamen of *PINK1*, *Parkin* and *SNCA* mutation associated PD.

**Figure 2:**
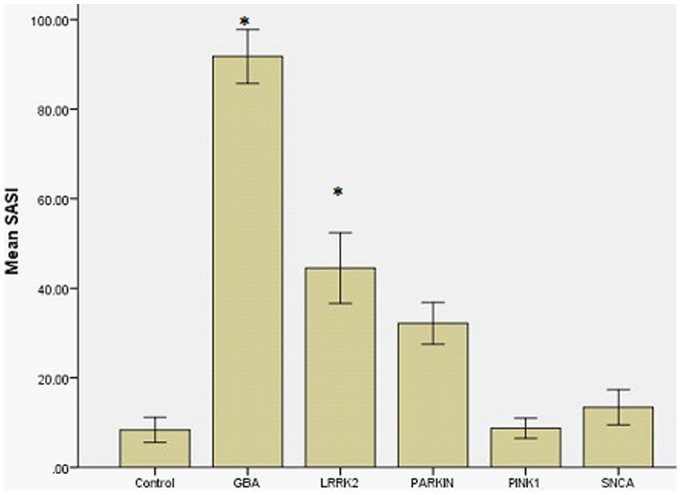
Striatal asymmetry index by genotype. The striatal asymmetry index was significantly higher for the GBA and LRRK2 genotypes (p<0.001). Graph shows mean SASI +/−1 standard error.

**Figure 3:**
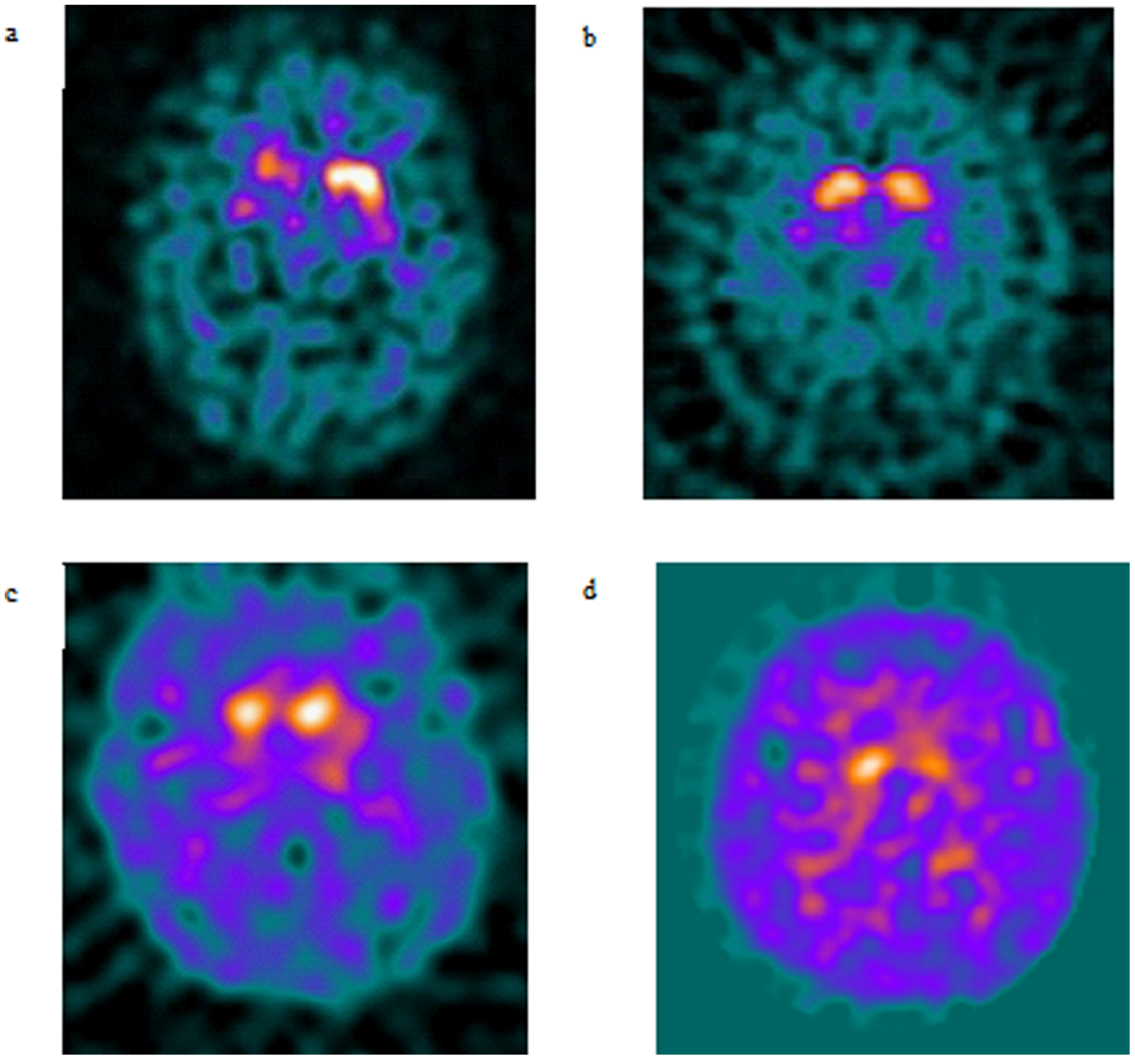
Representative DaTSCAN images. 3a. DaTscan from 50 year old man with *GBA* mutation, note predominantly right sided tracer loss. 3b. DaTscan from 67 year old woman with PINK1 mutation associated Parkinson's disease, note symmetrical loss of tracer uptake in caudate heads. 3c. DaTscan from 45 year old man with Parkin mutation associated Parkinson's disease and symmetrical loss of tracer uptake. 3d. DaTscan from 32 year old man with LRRK2 mutation, note asymmetrical loss of tracer uptake in caudate heads.

To further explore these findings, the whole striatum asymmetry index (SASI) was calculated (Figure 2). Mann-Whitney U testing with bonferroni correction demonstrated that the SASI for PD-GBA (mean 80.5+/−5.1, p = 0.001) and LRRK2 (44.0+/−11.5, p = 0.009) was significantly higher than controls (mean 8.3+/−2.7). The SASI did not differ significantly between *PINK1*, *Parkin*, *SNCA* and controls (p>0.05 for all). Representative DaTSCANS are shown in Figure 3.

## Discussion

Here we report what is, to our knowledge, the largest series directly comparing the dopaminergic imaging features of the most important causes of genetic PD. The key finding is a difference in the pattern of imaging abnormality between the separate mutations. *GBA* and *LRRK2* mutations are associated with relatively asymmetric striatal dopaminergic neuronal loss, while *Parkin, PINK1* and *SNC*A mutation patients have relatively symmetrical loss. We believe that this difference in the pattern of striatal dopaminergic loss reflects separate influences of the respective mutations on the aetiopathogenesis of PD.

The results of our comparison of dopaminergic neuronal imaging features are in keeping with previous reports of imaging of single PD genotypes. There are 2 reports of DAT-SPECT in Gaucher disease patients with PD or PD patients with heterozygous *GBA* mutations [16], [17]. Though not formally quantified, inspection of the published images reveals clear asymmetry of radioligand uptake affecting the striatum. Here we confirm asymmetric loss of dopaminergic neurons in PD associated with heterozygous *GBA* mutations.

Multiple studies of *Parkin* mutation associated PD using both DAT-SPECT and 18-F-DOPA-PET clearly describe a symmetrical reduction in striatal radioligand uptake [18]–[20]. The DAT-SPECT and 18-F-DOPA-PET features of PD patients with bi-allelic *Parkin* mutations reported herein is entirely in keeping with this. DAT-SPECT in *PINK1* linked PD is usually described as symmetrical but some reports state that it is asymmetrical in a pattern reminiscent of sporadic PD [9], [21]–[22]. As previously described we found that the *LRRK2* mutation carriers with PD had relatively asymmetric loss of radioligand uptake on DAT-SPECT [23], [24]. The *SNCA* mutation PD cases reported here had symmetric reduction of ligand binding. This is in keeping with reports of 18F-DOPA in PD with the A53T mutation which demonstrated relatively symmetrical loss of radioligand uptake [25], however, reports of *SNCA* multiplication associated PD describe an asymmetrical pattern of reduced tracer uptake [26], [27].

The currently available data on the pathogenetic mechanisms underlying monogenetic PD may help to explain the differences in symmetry of radioligand uptake between genotypes [28], [29]. Intuitively, it might be expected that a genetic cause of PD present from birth and presumably expressed symmetrically in expressing tissues, would result in symmetric clinical features and striatal imaging loss. In agreement with this *PINK1* and *Parkin* mutations are generally associated with early onset, relatively symmetrical parkinsonism [1], [6]. Both *PINK1* and *Parkin* proteins are proposed to play a role in clearing damaged mitochondria, and there is evidence that loss of function of these proteins is associated with mitochondrial dysfunction [30], [31]. The diffuse and symmetrical loss of dopaminergic neurons reported by us, and others [18], [20]–[22], may reflect widespread dysfunction and degeneration of striatal dopaminergic neurons due to a general predisposition to neuronal mitochondrial dysfunction in *PINK1* and *Parkin* mutation associated PD. *SNCA* mutations are proposed to act chiefly by facilitating Lewy body formation [32]. The symmetrical loss of dopaminergic neurons on DAT-SPECT reported herein may thus reflect a generalised, homogeneous increase in Lewy body formation in the striatum since the mutation is present in every cell.

By contrast, *GBA* and *LRRK2* mutations are most commonly associated with a phenotype and age of onset that closely resembles late onset sporadic PD [1], [4], [12]. This suggests that these mutant proteins may interact with additional pathogenetic process, either genetic or environmental, predisposing to age related PD. For example in PD with mono- or bi-allelic *GBA* mutations there is reduction or loss of glucocerebrosidase enzyme activity in the brain, most severe in the *substantia nigra* [35], associated with elevated alpha-synuclein deposition as measured by Western blot [35]. Both cell biology and mouse studies indicate that inhibition of glucocerebrosidase activity is associated with elevated alpha-synuclein accumulation [33]. The asymmetric loss of dopaminergic neurons observed in PD with *GBA* mutations may thus reflect a stochastic element whereby there is a focal loss of glucocerebrosidase activity below a critical level eventually resulting in an initially focal accumulation of alpha-synuclein and Lewy body formation. There is evidence from human brain and cell models that alpha-synuclein accumulation results in inhibition of glucocerebrosidase, thus causing a “feed forward” mechanism which could contribute to the spread of pathology and neurodegeneration [33], [34]. We recently described loss of glucocerebrosidase activity associated with alpha-synuclein accumulation in brain tissue from PD without *GBA* mutations [33]. This suggests that a similar mechanism may operate in sporadic PD and may account partly for the similarities in dopaminergic neuronal imaging and clinical phenotype between sporadic PD and PD associated with *GBA* mutations. We hypothesised that GBA mutations accelerated or enhanced pathogenetic mechanisms associated with the cause of sporadic PD [33] and the present imaging results support this. Mutant LRRK2 protein has recently been shown to inhibit chaperone mediated autophagy. In theory this could result in focal loss of lysosomal degradation of alpha-synuclein in the brain of *LRRK2 G2019S* mutation carriers, causing initially asymmetrical Lewy body deposition and loss of dopaminergic neurons [35].

Recessive forms of genetic PD (*Parkin*, *PINK1*) generally demonstrate relatively symmetric loss of radioligand uptake in the striatum. PD associated with heterozygous mutations in *GBA* or *LRRK2 G2019S*, which can present with dominant or apparently sporadic PD, produces a relatively asymmetric pattern of loss of radioligand uptake. In clinical practice asymmetric loss of radioligand binding should not exclude a genetic aetiology for PD while relatively symmetric decrease should raise suspicion of a monogenetic form of PD. The asymmetric loss of dopaminergic neurons in *GBA* and *LRRK2* associated PD supports the hypothesis of additional interactions with genetic or environmental factors leading to age related neurodegeneration in these genotypes.
